# Cluster Analysis of Categorical Variables of Parkinson’s Disease Patients

**DOI:** 10.3390/brainsci11101290

**Published:** 2021-09-29

**Authors:** Renee Hendricks, Mohammad Khasawneh

**Affiliations:** Department of Systems Science and Industrial Engineering, Watson College of Engineering and Applied Science, Binghamton University, New York, NY 13902, USA; mkhasawn@binghamton.edu

**Keywords:** cluster analysis, Parkinson’s disease, patient subtypes

## Abstract

Parkinson’s disease (PD) is a chronic disease. No treatment stops its progression, and it presents symptoms in multiple areas. One way to understand the PD population is to investigate the clustering of patients by demographic and clinical similarities. Previous PD cluster studies included scores from clinical surveys, which provide a numerical but ordinal, non-linear value. In addition, these studies did not include categorical variables, as the clustering method utilized was not applicable to categorical variables. It was discovered that the numerical values of patient age and disease duration were similar among past cluster results, pointing to the need to exclude these values. This paper proposes a novel and automatic discovery method to cluster PD patients by incorporating categorical variables. No estimate of the number of clusters is required as input, whereas the previous cluster methods require a guess from the end user in order for the method to be initiated. Using a patient dataset from the Parkinson’s Progression Markers Initiative (PPMI) website to demonstrate the new clustering technique, our results showed that this method provided an accurate separation of the patients. In addition, this method provides an explainable process and an easy way to interpret clusters and describe patient subtypes.

## 1. Introduction

Parkinson’s disease (PD) is a disabling and progressive disease, and it is prevalent in the ageing population [1]. James Parkinson first described PD in 1817, in a publication titled, “An Essay on the Shaking Palsy”, distinguishing between tremors at rest and during motion [1]. The main pathological feature of PD is the degeneration of neuromelanin-containing neurons and the presence of or increase in Lewy bodies (LBs), densely packed proteins, in the remaining neurons and other areas of the central nervous system [1]. Furthermore, the cause of the degeneration is unknown but it results in a loss of dopamine. Men may be 1.5 times more likely to be diagnosed with PD, with other possible risk factors including a family history of Parkinson’s disease, environmental factors, and even personality traits. People with a family history of the disease may have twice the risk [1]. PD diagnosis is based on clinical examination to determine whether any of the four motor symptoms are present: tremors at rest, rigidity, bradykinesia, and postural instability, with bradykinesia the most disabling feature that affects everything from fastening buttons to handwriting to the stopping of one or both arms swinging while walking, whereas tremors are involuntary movements caused by muscle contractions, which are the presenting feature in most cases [1].

Standardized rating scales attempt to quantify disease progression and severity, but these are based on interpretation, are subjective, and are based on an ordinal value. The surveys contain successive categories to choose from, but successive categories do not represent equal differences of a measured attribute; hence, the resulting data are ordinal and categorical [2]. Because these scales are ordinal in type, their resulting scores are nonlinear values, not providing a quantifiable progression or severity level, even though past cluster studies utilize these scale results to define and divide disease progression and severity levels among patients for a multitude of symptoms. Cluster analysis utilizing categorical variables, including demographic information, such as family history, and clinical patient information, such as symptom presence, may provide a method to accurately and clearly define PD patient subtypes.

## 2. Cluster Analysis

Cluster analysis divides data into groups (or clusters) that are meaningful, useful, or both, and groups data based on information found only in the data that describe the objects and their relationships [3]. Furthermore, the goal is that the objects within a group are similar to one another and different from the objects in other groups. Hence, the greater the similarity within a group and the greater the difference between groups, the better and more distinct the clustering [3].

However, the concept of a cluster is not well defined [3]. A cluster can be considered an ordered list of objects, with some common characteristics, where the objects belong to an interval of values, such as an interval [a, b] [4]. In addition, the distance (or measure) between clusters can involve some or all elements of the clusters, where the clustering method determines how the distance (s) should be computed.

Cluster algorithms are separated by different techniques and include hierarchical, partitioning, density-based, and model-based, utilizing probabilistic, distance-based, and dimensionality-reduction techniques [5]. The two most common types are hierarchical and partitioning algorithms. Hierarchical (non-partitioning) cluster methods can be agglomerative, where all points are individual clusters at the starting point and, at each step, the closest pair of clusters are merged [3]. This step is repeated until all data points are linked together and one large cluster emerges [6]. In divisive, hierarchical clustering, this method starts with one, all-inclusive cluster and, at each step, a cluster is split until only singleton clusters of individual points remain [3]. In partitioning cluster analysis, data are divided into non-overlapping subsets, where each data instance is assigned to one exactly subset [6]. Partitioning methods are useful for bioinformatics applications where a fixed number of clusters is desired; however, a drawback of this is that a user specifies the number of clusters as an input parameter [5].

A commonly utilized, partitioning clustering approach is called K-means clustering, in which data points, such as patients, are assigned to a prespecified (K) number of clusters without a hierarchical structure [7]. K initial clusters are formed, after which patients are assigned to the cluster they most resemble. Cluster means are calculated and the distance to each cluster mean is calculated for each patient. Patients are reassigned to another cluster if they are closer to that cluster mean than to the mean of the cluster originally assigned [7]. The cluster means are recalculated followed by calculation of the patients’ distances to the cluster means. This iterative process stops when no patients need to be reassigned [7].

A disadvantage of the K-means algorithm is that it may not provide the same result each time because the resulting clusters depend on initial, random assignments [4]. If the initial seeding positions are not chosen correctly, the clustering result will be adversely affected [6]. The K-means method does take into consideration data distribution [8]. A dataset can contain a subset that is larger in size than the other subsets, making the larger subset of higher importance or weight when the method is applied. In addition, K-means has trouble clustering data that contains outliers [3]. If outliers are present in the dataset, the cluster centers may not be as representative as they should be, and updating these center points incrementally introduces an order dependency, in which the resulting clusters depend on the order in which the points are processed [4]. In fact, there is nothing inherent in the K-means algorithm that guarantees true clusters will be discovered [9].

## 3. PD Patient Cluster Analysis Research

The intent of PD cluster analysis research is to understand the similarities and differences among patients, which may lead to patient subgroups that can assist with future diagnosis, as well as symptom and progression tracking and treatment. A systematic review of PD patient clustering research was conducted in [10], describing and critiquing the variables included in clustering, the cluster methods applied, the resulting patient subgroups, and the evaluation metrics. The majority of studies included a variety of clinical scale scores for clustering, which provide a numerical, but ordinal, categorical value, even though these values were treated as numerical variables, which was incorrect. In addition, categorical variables, such as biological sex and family history, which may also provide useful insights into disease diagnosis, progression, and treatment, were excluded from the clustering. This may be because the most common clustering method applied, K-means, is only applicable to numerical values, as it applies a distance measurement to cluster variables.

Furthermore, past cluster results pointed to two to five patient clusters; these values were predefined by the end users, with similarities among the clusters in regard to patient ages and disease durations, pointing to the possibility of excluding these limited-range values in future cluster analyses. The studies also lacked the use of existing clustering evaluation metrics to evaluate the separation of the resulting clusters [10].

## 4. Materials and Methods

**Figure 1:**
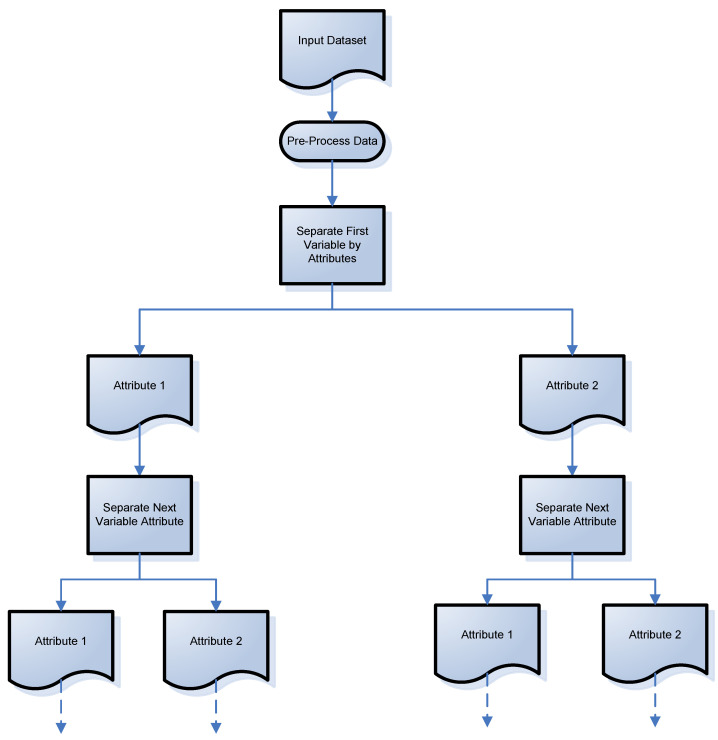
Universal discovery tree clustering concept (patent pending).

Given the limitations of existing clustering algorithms, a universal clustering discovery tree is proposed. The patent is pending on this method. It is universal because it can handle a multitude of data types including categorical (numerical or text format), numerical, and mixed datasets. Discovery refers to this method’s way of automatically discovering clusters without any input or guess by a user as to how many clusters may exist, in order for the clustering to occur. In this tree-line approach, each variable is separated by its attributes, one at a time. The process of this approach can be viewed in Figure 1. Starting with the first variable, there are two attributes that are separated out. Then, the next variable attributes are separated out, again showing a two-attribute variable, but any number of attributes can be evaluated with this method. The discovery tree continues until the last variable’s attributes are separated out.

The discovery tree method was developed so that variable order will not affect the final cluster results and provide the same result every time; this is a major limitation of existing clustering algorithms, as discussed earlier. A larger variable subset, such as a larger subset of males compared to females in the dataset, will not negatively affect the cluster result; another limitation of existing methods. The method automatically discovers the total number of clusters, eliminating the need for end-user input, making this a true, unsupervised method. This method can cluster all types of variables, including categorical, discrete, text, and mixed datasets. For continuous, numerical variables, the proposed method provides two conversion methods. The end user can convert the continuous variables to discrete numbers or categorical sets, prior to clustering. To highlight how this proposed method works, its application to categorical variables of a Parkinson’s disease patient dataset is explored in the results section. Descriptive, statistical analysis of the numerical variables will be applied to the largest patient clusters.

The results of this clustering method can be evaluated with existing, clustering metrics. One of these metrics is the silhouette coefficient, which is an unsupervised measure that incorporates both cohesion and separation [3]. It is also referred to as silhouette score or index and it is calculated as follows [3]: For the *i*th object, calculate its average distance to all other objects in its cluster; this is *a_i_*. For the *i*th object and any cluster not containing the object, calculate the object’s average distance to all the objects in the given cluster. Then, find the minimum value with respect to all clusters; this is *b_i_*. For the *i*th object, the silhouette coefficient is *s_i_* = (*b_i_* − *a_i_*)/max (*a_i_*, *b_i_*) [3]

The silhouette score can lead to values within the (−1, 0, 1) range, where a value close to −1 means the objects are poorly clustered, a value close to 1 means the objects are tightly clustered, and a value equal to zero indicates an indifferent case [11]. An overall measure of the goodness of a clustering method can be obtained by computing the average silhouette coefficient of all points [3].

The Parkinson’s Progression Markers Initiative (PPMI) was initiated to identify biomarkers of Parkinson’s disease progression through biologic sampling and clinical and behavioral assessments. PPMI is taking place at clinical sites in the United States, Europe, Israel, and Australia. Data and samples from study participants will enable the development of a comprehensive Parkinson’s database and biorepository available to the scientific community [12]. The PPMI dataset utilized for this study contained 423 patients, of whom 277 (65.5%) were male and 146 (34.5%) were female. In total, 19 of the 423 patients (4.5%) were under the age of 40 (25–39 years of age). The median disease duration was 4.3 months, with a range of weeks to 35.8 months (less than 2 years). In this dataset, 75% of patients did not have a family history of PD. In addition, 234 patients (55.3%) had motor symptoms that affected the right side of their body whereas 179 (42.3%) had symptoms that affected the left side of their body, with 10 (2.4%) with symptoms that affected both sides of their body. In terms of motor symptoms present at diagnosis, 78.3% had tremor symptoms, 75.6% had rigidity symptoms, and 82.3% had bradykinesia symptoms. The patient number variable was retained as this was a metadata point that referred back to the patient in the spreadsheet. The variables retained for analysis are displayed in Table 1.

PD patients with symmetrical symptoms were removed from the study because of the small percentage (2.4%) of the total. One patient was removed because there was a missing data point for family history, retaining a total number of 412 patients for cluster analysis. The information on this dataset, after pre-processing, is in Table 2. Median disease duration remained the same as the original dataset at 4.3 months, with the same range. Of the 412 patients, 76% (312) did not have a family history of the disease, whereas 24% (100) had a family history of the disease. In addition, 233 patients (55.3%) had motor symptoms that affected the right side of their body whereas 179 (42.3%) had an effect on the left side of their body. In terms of motor symptoms present at diagnosis, the percentages remained the same.

## 5. Results

This section provides the results of the universal clustering discovery tree applied to the six categorical variables of the PPMI dataset. The two numerical variables of age of onset and disease duration were analyzed during the post-analysis of the cluster results. Starting with the male (0) and female variable attributes (1), these were divided into two distinct groups. The next variable, family history, was also split into two groups because of the two attributed values (with a family history of PD and no family history of PD), under each of the male and female divisions. This separation continued with the remaining categorical variables’ attributes: dominant (symptom) side, symptom 1 (resting tremor), symptom 2 (rigidity), and symptom 3 (bradykinesia), present at disease diagnosis.

**Figure 2:**
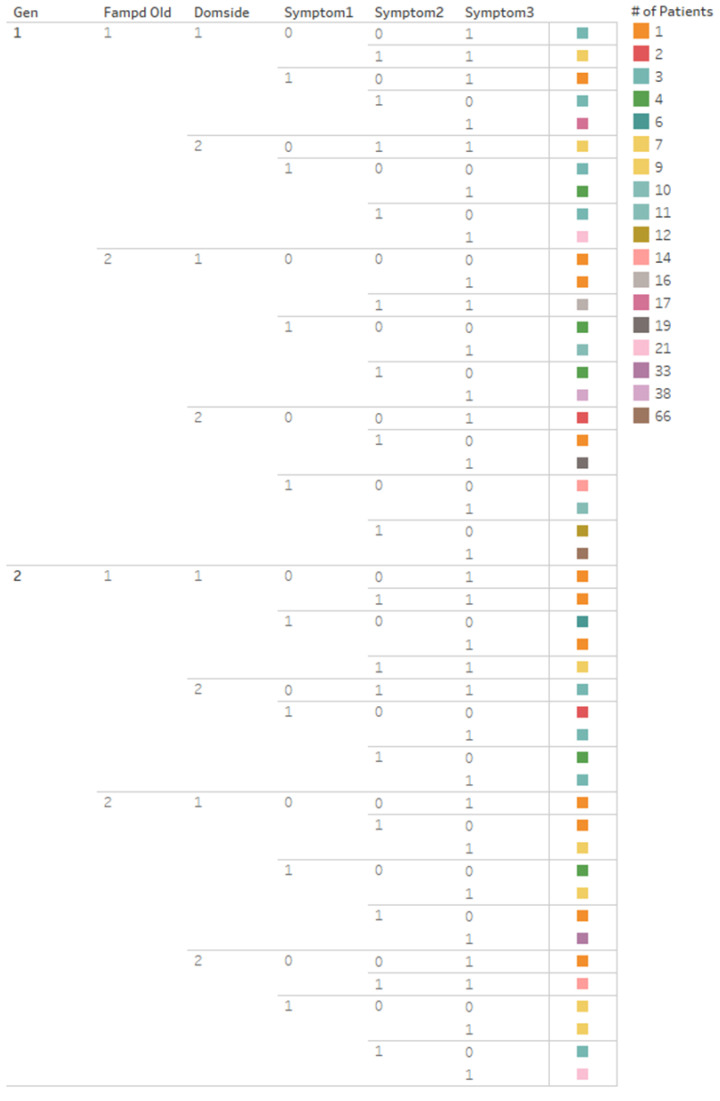
Universal clustering discovery tree results with PPMI categorical variables (in Tableau^®^).

Utilizing Tableau’s plotting functions to display the result, each variable was selected and placed into the plotting space, starting with the biological sex variable. From the 412 PD patients, 47 clusters were automatically discovered, ranging from 1 patient up to 66 patients. The results are in Figure 2. The division of variable attributes can be viewed. The patient clusters, which are listed per row, can be described by reciting each attribute value. For example, cluster one (row one) contains male patients (1) with a family history of PD (1), affected on the left side (1), and with symptom 3 (bradykinesia, 1) present at diagnosis. This cluster also contained three patients as per the orange color coding.

Tableau results were exported into Excel, with the cluster results listing an increasing number of patients, as viewed in Table 3. The cluster outlined in blue in row five contained one patient who did not have any of the three motor symptoms present at diagnosis. This may be a possible outlier. Reviewing the original PPMI spreadsheet, there was a notation that this patient had the motor symptom, gait difficulty.

In addition, the clusters with the largest number of patients, the five bottom rows, contained patients who had all three motor symptoms. Further reviewing the clusters where patients had all three motor symptoms present at diagnosis, eight clusters, outlined in orange, were discovered. These eight clusters contain exactly 50% of the entire dataset, with 206 of the 412 patients. The four largest clusters of males with all three motor symptoms equaled 142 out of 268, i.e., greater than half of the male subset at 53%. The four largest female clusters with all three symptoms contained 64 patients of 144, i.e., less than half of the female subset at 44%.

**Figure 3:**
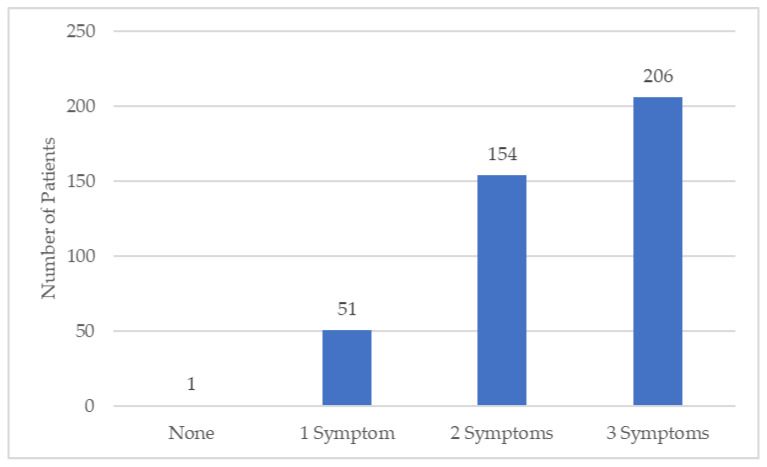
PD patients and number of motor symptoms.

There was a total of 15 clusters containing 51 patients (12% of the patients) who had 1 motor symptom and 23 clusters with 154 (37%) patients who had 2 motor symptoms. The clusters with patients with 1 motor symptom included 9 patients with bradykinesia, 2 patients with rigidity, and 40 patients with tremors, present at diagnosis. The clusters with patients with 2 motor symptoms included 76 patients with rigidity and bradykinesia, 48 patients with tremors and bradykinesia, and 30 patients with tremors and rigidity. As the number of symptoms increased, the number of patients increased in this dataset, as viewed in Figure 3.

Descriptive statistical measures were calculated for the five largest categorical cluster results from the universal discovery tree method. The largest male cluster contained 66 patients and the largest female cluster contained 33 patients, as shown in Table 4. The age of onset and disease durations were similar among the clusters, with age of onset median ranging from 59.5 to 61.4 years and disease duration median ranging from 3.0 to 4.3 months. This aligns with previous cluster analysis results and is expected as the age of onset for PD tends to be a limited age range, from 60 years of age and older, with a limited number of younger patients. This set of patients also had limited disease durations of less than 3 years, hence the similarities among durations in the five largest clusters. This supports the need to include categorical variables for PD patients and exclude variables that are similar among the population or original dataset.

Previous PD patient cluster studies did not utilize nor report silhouette scores of the cluster results. As mentioned earlier, an average silhouette score is commonly utilized for evaluating cluster results. Cluster validity measures tend to define cohesion, separation, or a combination of these, and can be applied to overall cluster results and individual clusters. The silhouette score incorporates both cohesion and separation [3]. A high silhouette score points to similarities among the data points within the clusters.

**Figure 4:**
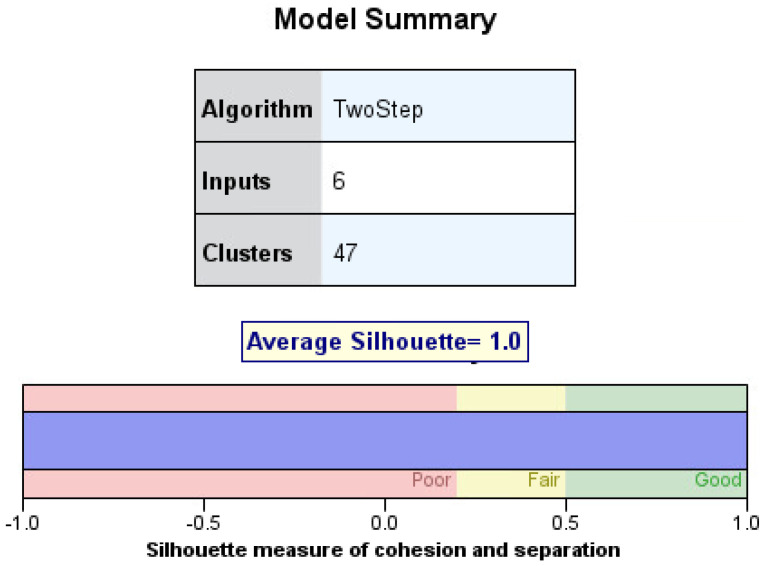
Average silhouette score of universal clustering discovery tree.

Utilizing the software program, IBM^®^ SPSS Statistics, for silhouette score calculation, a high score of 1.0 was computed for the 47 discovered clusters, as seen in Figure 4. This demonstrates the high accuracy of this method when applied to a categorical dataset. This result is expected because the variable attributes are creating the splits, meaning no attribute will be placed in an incorrect cluster, and the patients in each cluster have identical attributes.

## 6. Discussion

Past PD cluster analysis studies utilized clinical scales to define motor and non-motor symptoms’ presence and severity; however, these scores do not define disease progression nor severity in a quantifiable way but provide an ordinal, classification result. In addition, a set of these studies defined and calculated disease progression by dividing the scale scores by the time since diagnosis, which is incorrect and does not convert the scale score(s) to a quantifiable value. Cluster analysis needs to be conducted with accurate patient demographics, disease symptoms, and treatment results. In addition, past studies did not utilize the categorical variables of biological sex, family history, or dominant body side affected by the disease in the cluster analysis, providing a new way to describe patient subtypes.

The majority of past studies utilized K-means clustering, a common method where a predefined number of clusters is inputted prior to analysis. How the number of clusters was chosen was not provided in some of the studies. This selection is important, as an inaccurately chosen value will provide inaccurate patient assignments. In some studies, K values were based on a previous study and not on the dataset under review; this is incorrect. In addition, the K-means method, which was commonly applied in past cluster studies, includes a series of limitations, including not yielding the same result with initial, random assignments [4], not taking into consideration the data distribution [8], and being unable to handle data outliers [3].

In past PD cluster studies, silhouette scores were not reported. An average silhouette score is commonly utilized for evaluating cluster results. A high silhouette score points to similarities among the data points within the clusters. Future studies with a rigorous design, standardized with respect to the included variables, data processing, and clustering analysis technique, may advance the knowledge of PD subtypes [7].

## 7. Conclusions

This paper outlined a universal clustering discovery tree applied to a categorical dataset. This study utilized a PD patient dataset from the Parkinson’s Progression Markers Initiative (PPMI) website to demonstrate the proposed clustering technique and to test its accuracy on the categorical variables. A total of 47 clusters were discovered with the 6 categorical variables. These results provided a perfect, average silhouette score of 1.0. As with any method, end users have to balance accuracy with results management; for end users who are looking for the most accurate method, which may provide a large number of resulting clusters, this will be the method of choice. A large number of clusters does not point to a weakness in this method but instead to the diversity that may occur among patients and disease symptoms. In addition, this clustering method is simple to use for medical practitioners and researchers, and its results are interpretable and explainable. The next steps are to automate this new clustering technique for widespread ease of use. The expectation of further use of this method is that it will provide a direction for treating Parkinson’s disease patients, with a focus on personalized medicine, treatment in clusters, or a mixture of both applications.

## 8. Patent

The universal, clustering discovery tree proposed is patent pending.

## Figures and Tables

**Table 1:** PPMI variables retained for analysis.

**Variable Name: Meaning**	Ageonset: Age at PD diagnosis
PATNO: Patient Number	DOMSIDE: Side most affected by PD at onset
Gen: Gender (referred to as biological sex in the remaining text)	Symptom 1: Initial symptom at diagnosis: Resting tremor
Fampd_old: Family history of PD: Any family, No family history	Symptom 2: Initial symptom at diagnosis: Rigidity
Duration: disease duration since diagnosis	Symptom 3: Initial symptom at diagnosis: Bradykinesia

**Table 2:** PPMI PD patient variables for cluster analysis.

Biological sex: 268 males (1), 144 females (2)
Family history of PD: 100 (1), no family history of PD: 312 (2)
Disease duration: 0.4–35.8 months, median: 4.3 months
Age of onset: 25.4–83.0 years, median: 60.3 years
Dominant symptom side: 179 left (1), 233 right (2)
Symptom 1 tremor (at diagnosis): 88 absent (0), 324 present (1)
Symptom 2 rigidity (at diagnosis): 98 absent (0), 314 present (1)
Symptom 3 bradykinesia (at diagnosis): 73 absent (0), 339 present (1)

**Table 3:** Universal clustering discovery tree results with PPMI categorical variables.

Gen	Fampd Old	Domside	Symptom 1	Symptom 2	Symptom 3	# of Patients
1	1	1	1	0	1	1
2	1	1	0	0	1	1
2	1	1	0	1	1	1
2	1	1	1	0	1	1
1	2	1	0	0	0	1
1	2	1	0	0	1	1
2	2	1	0	0	1	1
2	2	1	0	1	0	1
2	2	1	1	1	0	1
1	2	2	0	1	0	1
2	2	2	0	0	1	1
2	1	2	1	0	0	2
1	2	2	0	0	1	2
1	1	1	0	0	1	3
1	1	1	1	1	0	3
1	1	2	1	0	0	3
1	1	2	1	1	0	3
2	1	2	0	1	1	3
2	1	2	1	0	1	3
2	1	2	1	1	1	3
2	2	2	1	1	0	3
1	2	1	1	0	0	4
1	2	1	1	1	0	4
2	2	1	1	0	0	4
1	1	2	1	0	1	4
2	1	2	1	1	0	4
2	1	1	1	0	0	6
1	1	1	0	1	1	7
2	1	1	1	1	1	7
1	1	2	0	1	1	7
2	2	2	1	0	0	7
2	2	1	0	1	1	9
2	2	2	1	0	1	9
2	2	1	1	0	1	9
1	2	1	1	0	1	10
1	2	2	1	0	1	11
1	2	2	1	1	0	12
1	2	2	1	0	0	14
2	2	2	0	1	1	14
1	2	1	0	1	1	16
1	1	1	1	1	1	17
1	2	2	0	1	1	19
1	1	2	1	1	1	21
2	2	2	1	1	1	21
2	2	1	1	1	1	33
1	2	1	1	1	1	38
1	2	2	1	1	1	66

**Table 4:** Descriptive statistical measures for the five largest PD patient clusters.

Cluster #	Cluster #17	Cluster #24	Cluster #29	Cluster #41	Cluster #47
# of PD Patients	38	33	21	66	21
Biological Sex	Males	Females	Males	Males	Females
Age of Onset (Years)	59.5 Median 9.4 Std. Dev.	58.5 Median 10.0 Std. Dev.	58.8 Median 9.8 Std. Dev.	61.4 Median 9.9 Std. Dev.	59.1 Median 7.5 Std. Dev.
Disease Duration (Months)	3.8 Median 5.2 Std. Dev.	4.0 Median 6.7 Std. Dev.	3.0 Median 5.1 Std. Dev.	4.1 Median 6.8 Std. Dev.	4.3 Median 3.6 Std. Dev.

## Data Availability

Data used in the preparation of this article were obtained from the Parkinson’s Progression Markers Initiative (PPMI) database (www.ppmi-info.org/data, accessed on 1 February 2020). For up-to-date information on the study, visit www.ppmi-info.org, (accessed on 1 January 2021).
